# Effects of Short Chain Fatty Acid-Butyrate Supplementation on the Disease Severity, Inflammation, and Psychological Factors in Patients With Active Ulcerative Colitis: A Double-Blind Randomized Controlled Trial

**DOI:** 10.1155/jnme/3165876

**Published:** 2025-03-16

**Authors:** Donya Firoozi, Seyed Jalil Masoumi, Seyed Mohammad-Kazem Hosseini Asl, Mohammad Fararouei, Sanaz Jamshidi

**Affiliations:** ^1^Student Research Committee, School of Nutrition and Food Sciences, Shiraz University of Medical Sciences, Shiraz, Iran; ^2^Nutrition Research Center, School of Nutrition and Food Sciences, Shiraz University of Medical Science, Shiraz, Iran; ^3^Gastroenterohepatology Research Center, Shiraz University of Medical Sciences, Shiraz, Iran; ^4^Department of Internal Medicine, Gastroenterology Ward, School of Medicine, Shiraz University of Medical Sciences, Shiraz, Iran; ^5^Department of Epidemiology, School of Public Health, Shiraz University of Medical Sciences, Shiraz, Iran; ^6^Center for Cohort Study of Shiraz University of Medical Sciences Employees' Health, Shiraz University of Medical Sciences, Shiraz, Iran

**Keywords:** anxiety, depression, inflammation, short-chain fatty acids, sodium butyrate, ulcerative colitis

## Abstract

**Background:** Depression and anxiety are common in UC patients due to gut microbiota dysbiosis and increased proinflammatory markers. Butyrate, a short-chain fatty acid, participates in the regulation of gut microbiota and inflammation and has neuroprotective effects in neurodegenerative disease. Therefore, we assessed the effects of sodium butyrate supplementation on the disease severity, inflammation, and psychological factors in active UC patients.

**Methods:** This study was a randomized, parallel, double-blind controlled trial. Participants in the intervention (*n* = 18) and control (*n* = 18) groups received 600 mg/kg of sodium butyrate or rice starch as a placebo with their main meal, respectively, for 12 weeks. The partial Mayo score was used to evaluate disease severity, while the Westergren method was employed to assess the erythrocyte sedimentation rate (ESR). NLR and PLR were determined using an automated analyzer (XS-500i, Sysmex). Moreover, the psychological factors were assessed by the hospital anxiety depression scale (HADS) and the general health questionnaire (GHQ).

**Results:** In comparison with placebo, sodium-butyrate supplementation significantly decreased the ESR level (−6.66 ± 1.56 vs. 3.00 ± 2.11, *p*=0.01), NLR (−0.24 ± 0.1 vs. 0.33 ± 0.23, *p*=0.02), Mayo score (−2.33 ± 0.41 vs. 0.22 ± 0.40, *p* < 0.001), HADS anxiety score (−2.77 ± 0.64 vs. 0.94 ± 0.63, *p*=0.001), HADS depression score (−2.38 ± 0.47 vs. 0.61 ± 0.33, *p* < 0.001), and GHQ total score (−12.11 ± 1.48 *vs*. 3.55 ± 1.39, *p* < 0.001).

**Conclusion:** Butyrate could serve as an effective adjuvant treatment for reducing disease severity and alleviating psychological symptoms. This trial was registered on the Research Ethics Committee of Shiraz University of Medical Sciences, with the reference number IR.SUMS.SCHEANUT.REC.1400.037.

**Trial Registration:** Iranian Registry of Clinical Trials: IRCT20211214053401N1

## 1. Introduction

Ulcerative colitis (UC) is a chronic inflammatory bowel disease (IBD) marked by an alternating period of relapse (active inflammation) and remission in the large intestine [1]. The symptoms of UC include inflammation, bloody diarrhea, cramps, and discomfort in the abdomen [2]. Additionally, mental symptoms such as depression and anxiety are common in UC patients [3], which have been linked to a decline in life quality, lower adherence to treatment, and higher morbidity and mortality [4]. According to a meta-analysis study, an estimated 32.6% of UC patients suffer from anxiety, and 23% suffer from depression. During the active phase of the disease, these amounts rise to 70.8% and 41.3%, respectively [5]. IBD has a bidirectional relationship with depression and anxiety through several mechanisms such as gut microbiome dysbiosis, increased proinflammatory cytokines, signaling from the vagal nerve, and changes in the brain morphology [6]. Moreover, immune-inflammatory responses happen in depression similar to IBD with common marker production, including an increase in the levels of proinflammatory cytokines such as interleukin (IL)-6, IL-1, IL-22, IL-17, interferon gamma (IFN-γ), tumor necrosis factor alpha (TNF-α), C-reactive protein (CRP), and Th-17-inducing cytokines [6, 7]. On the contrary, the production of transforming growth factor beta (TGF-β) and anti-inflammatory cytokines such as IL-10 decreased in both IBD and depression [8]. Additionally, the gut microbiome affects function and behavior due to communication with the brain, which directly impacts mental disorders. This communication occurs through the gut–brain axis, the hypothalamic–pituitary–adrenal (HPA) axis, immune system activation (mainly microglia in the brain), and the inflammatory response system [9]. Additionally, the microbiota of the gut produces neurotransmitters such as dopamine, γ-aminobutyric acid (GABA), and serotonin, which impact mood [10–12].

On the other hand, UC dysbiosis is associated with a reduction in gut bacteria that produce short-chain fatty acid (SCFA)-like clostridium and faecalibacterium, which are linked to both inflammation and depressive disorder [13, 14]. SCFAs (butyrate, acetate, and propionate) are produced from nondigestive carbohydrates via fermentation by gut microbiota in the lower intestine [15]. They play a pivotal function in the microbiota–gut–brain axis involved in IBD and depression's pathophysiology [16]. Also, SCFAs have anti-inflammatory and neuroprotective effects via complex biological mechanisms [17].

Butyrate is a crucial SCFA and the primary energy source of the colon. Its amount decreased in the intestine and stool of UC patients due to the dysbiosis described above [18]. Butyrate has the ability to modulate the dysbiosis occurring in gut microbiota [19] and anti-inflammatory effects through several pathways including suppression of nuclear factor kappa B (NF-κB), reducing the proinflammatory cytokine expression [20], activating the regulatory T-cells, and increasing the anti-inflammatory cytokines [21]. Moreover, butyrate exhibits neuroprotective effects by crossing the blood–brain barrier (BBB) in neurodegenerative disorders such as depression and anxiety [22]. It achieves these effects by reducing the activation of microglia and lipopolysaccharides [22, 23], acting as a HDAC inhibitor [24], increasing the level of brain-derived neurotrophic factor (BDNF) [25], promoting epigenetic modifications in the central nervous system (CNS) [22], causing neuroendocrine alterations [22], and having protective effects against chronic cerebral hypoperfusion (CCH) and neuroinflammation [26].

According to previous studies, approximately one-third of UC patients experiencing depression and two-thirds experiencing anxiety remain undiagnosed. On the other hand, the majority of UC patients with psychological disorders do not obtain a sufficient and effective treatment [27]. Therefore, assessing and treating depression and anxiety in UC patients are important for achieving better treatment responses and improving their quality of life. The aims of this research were to assess the effects of sodium butyrate supplementation on reducing disease severity and inflammation (as the sign of disease severity) and improving psychological factors including depression and anxiety in patients with active UC.

## 2. Materials and Methods

### 2.1. Study Design

The current parallel randomized double-blind, placebo-controlled clinical trial was conducted based on the Declaration of Helsinki for a 12-week period. Eligible patients were chosen from the IBD clinic located at Shahid Faghihi Hospital, Shiraz University of Medical Sciences, Shiraz, Iran. All participants provided informed consent, prior to their enrollment. Ethical approval of this study was obtained from the Research Ethics Committee of Shiraz University of Medical Sciences, with the reference number IR.SUMS.SCHEANUT.REC.1400.037.

### 2.2. Participants

36 participants (18 people in each butyrate and placebo group) were recruited for this trial according to the sample size estimation which was thoroughly explained in our previous study [28]. Inclusion criteria for this study were active UC patients with mild to moderate disease severity, confirmed based on histological and endoscopic assessments at least three months prior to the study's start. Additionally, patients had a body mass index (BMI) ranging from 18.5 to 30 kg/m^2^ and were between ages of 18 and 60. The exclusion criteria for this study included patients with any gastrointestinal, renal, autoimmune, cardiovascular diseases, cancer, and diabetes; use of anti-TNF-α, corticosteroids, and anti-inflammatory medications; use of any supplements such as multivitamin-mineral, pro-/pre-/syn-/postbiotics, omega 3, and antioxidants in the last three months; changes in dose or type of medications and disease severity or relapse during the study; alcohol and tobacco use during enrollment; COVID-19 infection two months prior to or during the trial; and pregnancy and lactation.

### 2.3. Randomization, Allocation Concealment, and Blinding

UC patients meeting eligibility criteria were randomly assigned to either the sodium butyrate or placebo based on block randomization with an allocation ratio of 1:1 and a block size of 4. A random number generator found at https://www.sealedenvelope.com/simplerandomiser was used for this assignment. This procedure was carried out by a statistician who was not involved in any other part of the trial. The randomization numbers were inserted into opaque, sequentially numbered envelopes by the third party to preserve allocation secrecy. When participants were admitted to the trial, these envelopes were opened sequentially. Thus, the participants, researchers, laboratory personnel, and result evaluators were blind to the allocated group until the database was opened. Notably, the butyrate and placebo capsules were packed in similar bottles labeled A and B, and they had the same color, size, and shape. Additionally, a tiny amount of butyrate was included in each placebo bottle to create a similar scent.

### 2.4. Intervention

Patients in the intervention group received 600 mg of sodium butyrate capsules, while those in the control group received 600 mg of rice starch capsules as a placebo, once daily with their main meals for 12 weeks. The sodium butyrate supplements were provided by Body Bio Company (Body Bio, USA). Each capsule contains 600 mg of butyric acid and other ingredients such as medium-chain triglycerides (MCT), hydroxypropyl methylcellulose, and sodium hydroxide. Sodium butyrate dosage was determined according to the recommendation guidelines of the manufacturer, which have been demonstrated as safe with no side effects based on prior studies [29]. Moreover, at the beginning of the trial, dietary recommendations specific for IBD were provided to participants according to their usual dietary routines.

### 2.5. Demographic, Anthropometric, and Dietary Assessments

At the beginning of the study, participants' general information, such as age, sex, medical history, disease extent and duration, marital status, alcohol consumption, and smoking, was collected through a questionnaire. At the start and end of the study, body weight measurements were taken by a Seca scale (Seca, Germany) with a 100 gr precision, while participants were wearing light clothing and barefoot. Height was measured with a wall-mounted height rod, with a precision of 0.1 cm, and participants were measured barefoot at the beginning of the study. The BMI was determined by dividing the weight in kilograms by the height in meters squared. Dietary intakes were assessed by a 3-day analog food recall (two regular and a weekend day) at the start and end of the trial. Nutritionist IV software (First Databank, San Bruno, CA, USA) modified for Iranian food was used for the determination of daily calorie and nutrient intake.

### 2.6. Disease Severity Assessment

The partial Mayo scoring index was utilized for assessment of UC severity at the start and end of the trial. This questionnaire contains three questions about frequency of stool, bleeding from the rectum, and the physician's overall assessment. Each question is scored between 0 and 3, with the overall score classified as follows: 0-1 represents remission, 2-4 represents mild disease, 5-6 represents moderate disease, and 7-9 represents severe disease. The study included participants with mild to moderate active UC based on their scores, which varied from 2 to 7 [30].

### 2.7. Blood Sampling and Biochemical Assessment

Fifteen milliliters of venous blood were taken from each participant at the start and end of the study after overnight fasting (10–12 h). The level of the erythrocyte sedimentation rate (ESR) was measured by the Westergren method. For the determination of the neutrophil to lymphocyte ratio (NLR) and the platelet to lymphocyte ratio (PLR), a Sysmex XS-500i automated analyzer (Sysmex, Japan) was used. The NLR was computed by divided count of neutrophil to count of lymphocyte, and the PLR was computed by divided count of platelet to count of lymphocyte.

### 2.8. Psychological Assessment

To screen for depression and anxiety, the Iranian validated version of the Hospital Anxiety and Depression Scale (HADS) was used at the baseline and end of the study by face-to-face interview. This questionnaire comprises seven questions for assessing anxiety and seven questions for assessing depression. Each question is scored between 0 and 3, with higher scores indicating a greater level of anxiety and depression, and the total score higher than 7 is considered as a cut-off point. Additionally, psychological distress among patients was assessed using the Iranian validated version of General Health Questionnaire-28 (GHQ-28) at the baseline and end of the study by face-to-face interview. This questionnaire consists of 28 items that evaluate symptoms related to anxiety, depression, social dysfunction, and loss of confidence. Each item is scored between 0 and 3 based on Likert scoring, with higher scores indicating higher levels of distress, and the total score of 23 is set as the cut-off point [31].

### 2.9. Compliance Assessment

Patients received weekly phone calls to monitor their compliance with supplement consumption. Furthermore, a visit was scheduled in the sixth week to check for general health status or any side effects and distribute the second round of supplements for the following 6 weeks. To assess the compliance rate, participants were requested to return any remaining capsules they had not consumed at the sixth and twelfth weeks. Participants were deemed compliant if they consumed at least 85% of the supplements provided.

### 2.10. Statistical Analysis

The Statistical Package for Social Sciences (SPSS) software (version 20.0, SPSS Inc., Chicago, IL, USA) was used for analysis of data based on the intention-to-treat (ITT) method. The qualitative and quantitative variables were reported as the number (percentage) and mean ± standard error, respectively. Normality of quantitative data was determined by the Shapiro–Wilk test and skewness and kurtosis statistics. Appropriate functions, such as logarithm (log10) and square root, were used to transform non-normally distributed data. An independent sample *t*-test was utilized to evaluate baseline patient characteristics and changes in nutritional intake between the two groups. Additionally, suitable methods such as Chi-square or Fisher's exact were employed for categorical data. The paired *t*-test was used for within-group comparisons. The mean difference (after-before) was computed for the between-group comparison variables, and the independent *t*-test was used to examine the mean differences between the two groups. The significance level was considered equal or less than 0.05.

## 3. Results

### 3.1. Participant Enrollment

**Figure 1:**
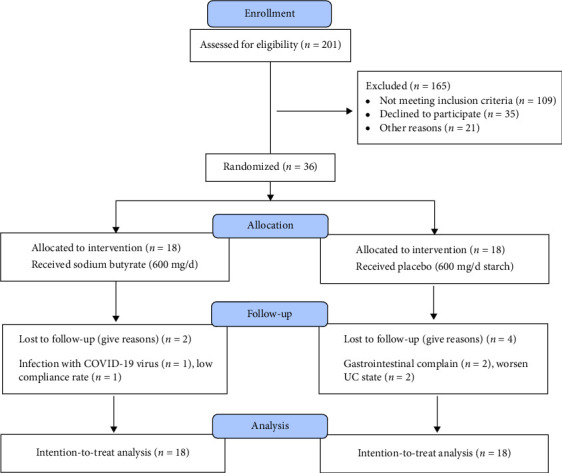
CONSORT flow diagram of participants during the trial.

In this clinical trial, which was conducted between January 2022 and February 2023, 201 patients were assessed for eligibility. Ultimately, 36 patients (20 men and 16 women) in the active phase with severity of mild to moderate met the inclusion criteria and were randomly assigned into either the intervention (*n* = 18) or placebo (*n* = 18) groups. During the trial, four patients dropped out in the placebo group due to worsening the disease (*n* = 2) and gastrointestinal complaints (*n* = 2), while two patients dropped out in the sodium butyrate group due to COVID-19 infection (*n* = 1) and a low rate of compliance (*n* = 1). However, based on the ITT principle, total of 36 participants were included for final analysis (Figure 1).

### 3.2. Demographic and Baseline Characteristics of Participants

According to Table 1, there were no significant differences between demographic and baseline characteristics of the patients in terms of age, sex, marital status, education, smoking habit, alcohol drinking, disease duration, current treatment, BMI (kg/m^2^), weight (kg), SBP, DBP, WBC, platelet count, ESR, hs-CRP, NLR, PLR, GHQ total, HADS depression, HADS anxiety, and partial Mayo score in the control and intervention groups. Furthermore, Table 2 shows that energy and nutrient intakes did not significantly differ between the two groups.

### 3.3. The Impact of Sodium Butyrate on the Inflammatory Markers

In this research, inflammatory markers including the ESR, NLR, and PLR as well as the partial Mayo score were evaluated for disease severity. Additionally, in our previous research, the level of calprotectin and hs-CRP were also assessed [28]. According to within-group analysis, the level of the ESR (*p* < 0.001) and the NLR (*p*=0.02) as well as the Mayo score (*p* < 0.001) significantly decreases in the sodium butyrate group before and after treatment, without any significant changes in the placebo group (Table 3). The between-group analysis revealed significant decreases in the levels of the ESR (*p*=0.01), NLR (*p*=0.02), and Mayo score (*p* < 0.001) in the sodium butyrate group compared with the placebo group. Additionally, while the PLR exhibited a decreasing pattern, it did not reach statistical significance (Table 3).

### 3.4. The Impact of Sodium Butyrate on the Psychological Factors

Participants in both groups were classified as having a psychological disorder based on a GHQ total score > 23 and HADS depression and anxiety scores > 7. In the sodium butyrate group, a significant reduction was observed in the HADS anxiety (*p* < 0.001), HADS depression (*p* < 0.001), GHQ somatic (*p* < 0.001), GHQ anxiety (*p* < 0.001), and GHQ total (*p* < 0.001), before and after treatment (Table 4). Additionally, the between-group analysis revealed significant decreases in the scores of HADS anxiety (*p*=0.001), HADS depression (*p* < 0.001), GHQ somatic (*p* < 0.001), GHQ anxiety (*p* < 0.001), and GHQ total (*p* < 0.001) in the sodium butyrate group compared with the placebo group over the 12-week intervention (Table 4).

## 4. Discussion

The present study performed on UC patients revealed the beneficial effect of sodium butyrate on disease severity, inflammatory biomarkers, and physiological factors. The remarkable influence of sodium butyrate was mainly observed in the decrease of the ESR and the NLR but not the PLR. It also reduced the partial Mayo score as an indicator of disease severity. Interestingly, sodium butyrate improves HADS anxiety and HADS depression indicators. In addition, sodium butyrate was closely associated with GHQ score reduction in somatic, anxiety, and total subcategories.

Our results support the findings from experimental studies that report anti-inflammatory activity of sodium butyrate. In this regard, Chen et al. have shown that sodium butyrate improved intestinal epithelium barrier integrity and inflammatory reaction by preventing AKT signaling pathways and NF-κB in the colitis mice model [32]. According to the animal model of UC, sodium butyrate administration ameliorated mucosa lesion and reduced the inflammatory response and leukocyte (neutrophil and eosinophil) infiltration [33]. These findings are consistent with the previous report by Liu et al. in the mice model with lipopolysaccharide-induced acute lung injury, which showed downregulation of NF-κB, toll-like receptor 4 (TLR4), IL1β, IL6, and TNF-α gene expression [34]. Similarly, in a diabetic-induced model in mice, oral butyrate supplementation attenuated inflammatory cytokines such as MCP-1, TNF-α, and IL-1β. Also, gut microbiome composition and gut epithelial barrier integrity were restored [35]. Moreover, oral administration of butyrate in patients with mild to moderate Crohn's disease induces clinical remission through ESR, NF-κB, and IL-1β reduction [36]. Furthermore, in a clinical trial by Roshanravan et al., hs-CRP and MDA biomarkers decreased significantly in participants with diabetes type 2 who receive sodium butyrate [37]. Additionally, a recent randomized clinical trial demonstrated that a butyrate-based formula improved the fatty liver index (FLI) and plasma lipid profiles in individuals with liver steatosis and metabolic syndrome [38]. In contrast, in a prospective observational study, there was no significant effect of sodium butyrate on the ESR and CRP; however, faecal calprotectin diminished remarkably in UC patients [39]. Moreover, elevated PLR and NLR values were reported in active UC patients [40], and a recent study showed reduction of the NLR family protein expression by sodium butyrate [41]. The PLR and NLR have been explored as an inexpensive, simple, and effective method for capturing inflammation [42], and based on our findings, both the PLR and NLR had decreasing trend in the sodium butyrate group; however, PLR changes were not significant. Similarly, a study in patients with COVID-19 revealed that the PLR and NLR can serve as a reliable predictor of survival/mortality [43]. Another study introduced NLR as an independent indicator of poor clinical outcomes [44].

The potential mechanisms explaining the protective effect of sodium butyrate in UC patients include direct and indirect pathways. Since sodium butyrate is a main fuel of the colonic mucosa, it modulates intestinal permeability and tight junctions [45, 46]. On the other hand, sodium butyrate promotes hepatic production of glutamine, whose immune modulating effects confirm its anti-inflammatory properties [47, 48]. Previously, it was also demonstrated that butyrate can prevent oxidative stress induced by LPS in mesangial cells [49]. It is important to mention that UC is accompanied by gut dysbiosis and a reduced gut microbial diversity [50]. Evidences reported that sodium butyrate could reconstruct the composition of the gut microbial profile [19, 51], thereby ameliorating UC symptoms. In addition, butyrate interferes in gene expression and chromatin release that reduces proinflammatory cytokine production, including IL1b, TNF, and IL6 [52]. Also, butyrate down-regulates inflammation through inhibiting availability of oxygen in the gut, stimulating anaerobic bacterial dominance, and decreasing pathobionts growth [53].

We found that the prevalence of anxiety and depression was reduced after oral supplementation with sodium butyrate in comparison to the control group. Although depression aspect of the GHQ score did not change significantly, somatic, anxiety, and total values were improved by sodium butyrate intake. There is evidence that the prevalence of mental illnesses including anxiety and depression is higher in UC patients compared to the general population, which can lead to quality of life reduction in UC patients [4]. This makes it necessary to introduce treatment solutions for this condition. A study by Qiu et al. investigates the effect of sodium butyrate administration in the mice model for 10 days, and the results indicated that sodium butyrate is such an agent that could be used to prevent depression onset and progression based on the forced swimming test, tail suspension test, and sucrose preference test [23]. This result was consistent with the previous experiment, which claimed that the treatment effect of sodium butyrate on depression was through reduction of neuro-inflammation genes [54]. Based on the results of another animal study, sodium butyrate was introduced as a mood stabilizer and reversed the manic-like and depressive-like behaviors [24]. Even more, sodium butyrate demonstrated antidepressant effects in chronic mild stress-treated rats and could be related to its influence on the neurochemical pathways attributed to depression such as tricarboxylic acid cycle and mitochondrial respiratory chain complex enzymes [55]. This suggests that sodium butyrate has a protective effect in the brain by neutralizing free radicals [56].

These findings emphasize the potential of sodium butyrate supplements as an adjunct in the treatment of UC. Sodium butyrate as a kind of HDAC inhibitor exhibits neuroprotective and anti-inflammatory effects against behavioral deficits [57]. Furthermore, based on the Sun et al. study, the sodium butyrate antidepressant effect seems to increase brain serotonin (5-HT) concentration, BDNF expression, and BBB modification in mice exposed to mild stress [58]. In addition, sodium butyrate is able to reverse the mitochondrial complex damage [55]. In line with our findings, proinflammatory cytokines upregulate by sodium butyrate, which may be a potential mechanism for its antidepressant effects [23, 59]. Additionally, gut microbiome disruptions have been associated with several neuropsychiatric disorders, especially depression. Recent studies suggest the microbiota–gut–brain axis as a bidirectional communication pathway in pathogenesis of various mental complications [60]. Butyrate may play a protective role against gut microbiota dysbiosis via remodeling gut bacterial composition. Also, butyrate can mediate the gut–brain axis through GLP-1 secretion by G protein-coupled receptor activation in the distal small intestine and colon, which has neuroprotective effects and inhibits neuroinflammation [61].

### 4.1. Strengths and Limitations

To our best understanding, this study is the first clinical trial that provides evidence for the beneficial effect of sodium butyrate on the psychological factors and inflammatory markers such as the NLR and PLR in UC patients. The study's limitations include a small sample size of UC patients, the lack of analysis of gut microbiota composition and fecal butyrate levels, and the omission of key inflammatory cytokines such as IL-6 and TNF-α as well as some potential diagnostic biomarkers for depression such as BDNF. These constraints primarily stem from insufficient funding.

## 5. Conclusion

To sum up, the results of the present study indicated that sodium butyrate supplementation improved disease severity, inflammatory biomarkers, and psychological symptoms in some aspects. In this regard, we suggest that sodium butyrate could be used in UC patients to improve life quality and modulate disease activation and progression. A further study with a larger sample size is warranted.

## Figures and Tables

**Table 1:** Demographic and baseline characteristics of participants.

	Groups		*p* value
	Sodium butyrate (*n* = 18)	Placebo (*n* = 18)	
Age (years)	41.16 ± 2.58	38.16 ± 2.91	0.44^a^
Sex *n* (%)			0.73^b^
Female	7 (38.90)	9 (50.00)	
Male	11 (61.10)	9 (50.00)	
Marital status *n* (%)			0.17^b^
Married	13 (72.20)	8 (44.4)	
Single	5 (27.80)	10 (55.55)	
Education *n* (%)			0.49^b^
Postdiploma	6 (33.33)	8 (44.44)	
Diploma or less	12 (66.67)	10 (55.56)	
Smoking habit *n* (%)			0.42^b^
Yes	5 (27.77)	3 (16.66)	
No	13 (72.22)	15 (83.33)	
Alcohol drinking *n* (%)			0.63^b^
Yes	3 (16.66)	2 (11.11)	
No	15 (83.33)	16 (88.88)	
Ulcerative colitis duration (years)	5.66 ± 0.98	4.02 ± 0.76	0.24^a^
Current treatment *n* (%)			0.73^b^
Asacole plus aminosalicylate	9 (50.00)	11 (61.10)	
Aminosalicylates	9 (50.00)	7 (38.90)	
BMI (kg/m^2^)	24.19 ± 1.01	24.34 ± 1.12	0.96^a^
Weight (kg)	68.77 ± 3.04	69.16 ± 3.28	0.95^a^
SBP (mmHg)	106.77 ± 2.26	106.44 ± 2.17	0.91^a^
DBP (mmHg)	75.77 ± 1.67	76.88 ± 1.78	0.65^a^
WBC count (10^9^/L)	8.29 ± 0.62	7.69 ± 0.53	0.53^a^
Platelet count (10^9^/L)	340.83 ± 18.61	314.72 ± 21.44	0.35^a^
ESR (mm/h)	22.66 ± 3.71	17.94 ± 2.67	0.31^a^
hs-CRP (mg/L)	4.36 ± 1.69	4.31 ± 0.88	0.28^a^
NLR	2.10 ± 0.24	1.97 ± 0.17	0.66^a^
PLR	154.75 ± 16.76	144.74 ± 14.03	0.65^a^
HADS depression	9.11 ± 0.97	9.77 ± 0.79	0.60^a^
HADS anxiety	10.61 ± 0.84	11.27 ± 0.91	0.59^a^
GHQ total	30.44 ± 1.60	30.33 ± 1.34	0.95^a^
Partial Mayo score	3.55 ± 0.30	3.66 ± 0.22	0.66^a^

*Note:* The data were reported as mean ± standard error and frequency (%) for quantitative and qualitative data. *p* values of ≤ 0.05 were considered statistically significant.

Abbreviations: BMI, body mass index; DBP, diastolic blood pressure; ESR, erythrocyte sedimentation rate; GHQ, general health questionnaire; HADS, hospital anxiety and depression scale; hs-CRP, high-sensitivity C-reactive protein; NLR, neutrophil/lymphocyte; PLR, platelet to lymphocyte ratio; SBP, systolic blood pressure; WBC, white blood cell.

^a^Obtained from the independent sample *t*-test for quantitative variables.

^b^Obtained from Chi-square or Fisher's exact test for qualitative variables.

**Table 2:** Mean differences in dietary intake of participants during 12 weeks of intervention.

	Sodium butyrate (*n* = 18)	Placebo (*n* = 18)	*p* value^a^
Energy (kcal/d)	−55.57 (482.07)	−37.42 (447.41)	0.90
Carbohydrate (g/d)	−16.10 (73.37)	−25.02 (79.68)	0.73
Protein (g/d)	7.59 (35.80)	11.00 (29.85)	0.76
Total fat (g/d)	−3.03 (35.21)	1.00 (31.98)	0.72
Total dietary fiber (g/d)	−0.14 (7.81)	−1.5 (10.03)	0.65

*Note:* The data were reported as mean ± standard error. *p* values of ≤ 0.05 were considered statistically significant.

^a^Obtained from independent sample *t*-tests.

**Table 3:** Comparisons within and between groups of inflammatory markers and Mayo scores after an intervention of 12 weeks.

		Sodium butyrate (*n* = 18)	Placebo (*n* = 18)	*p* value^b^
ESR (mm/h)	Pre	22.66 ± 3.71	17.94 ± 2.67	0.01
	Post	16.00 ± 2.66	20.94 ± 3.73	
	Change	−6.66 ± 1.56	3.00 ± 2.11	
	*p* value^a^	< 0.001	0.34	

NLR	Pre	2.10 ± 0.24	1.97 ± 0.17	0.02
	Post	1.86 ± 0.25	2.31 ± 0.31	
	Change	−0.24 ± 0.10	0.33 ± 0.23	
	*p* value^a^	0.01	0.27	

PLR	Pre	154.75 ± 16.76	144.74 ± 14.03	0.06
	Post	136.07 ± 12.43	165.00 ± 20.21	
	Change	−18.67 ± 10.54	20.26 ± 17.42	
	*p* value^a^	0.1	0.29	

Partial Mayo score	Pre	3.55 ± 0.30	3.66 ± 0.22	< 0.001
	Post	1.22 ± 0.24	3.88 ± 0.38	
	Change	−2.33 ± 0.41	0.22 ± 0.40	
	*p* value^a^	< 0.001	0.59	

*Note:* The data were reported as mean ± standard error. *p* values of ≤ 0.05 were considered statistically significant.

Abbreviations: ESR, erythrocyte sedimentation rate; NLR, neutrophil to lymphocyte ratio; PLR, platelet to lymphocyte ratio.

^a^Obtained from the paired sample *t*-test.

^b^Obtained from independent sample *t*-tests.

**Table 4:** Within and between-group comparisons of psychological factors at baseline and after 12 weeks of intervention.

Variables		Butyrate (*n* = 18)	Placebo (*n* = 18)	*p* value^b^
HADS anxiety	Pre	10.61 ± 0.84	11.27 ± 0.91	0.001
	Post	7.83 ± 0.60	12.22 ± 0.77	
	Change	−2.77 ± 0.64	0.94 ± 0.63	
	*p* value^a^	< 0.001	0.27	

HADS depression	Pre	9.11 ± 0.97	9.77 ± 0.79	< 0.001
	Post	6.72 ± 0.73	10.38 ± 0.74	
	Change	−2.38 ± 0.47	0.61 ± 0.33	
	*p* value^a^	< 0.001	0.34	

*GHQ scores*				
Somatic	Pre	12.16 ± 0.47	10.77 ± 0.40	< 0.001
	Post	5.10 ± 0.54	12.05 ± 0.86	
	Change	−7.00 ± 0.66	1.27 ± 0.64	
	*p* value^a^	< 0.001	0.06	
Anxiety	Pre	9.88 ± 0.87	9.27 ± 0.61	< 0.001
	Post	6.05 ± 0.33	10.66 ± 0.82	
	Change	−3.83 ± 0.80	1.38 ± 0.65	
	*p* value^a^	< 0.001	0.06	
Social	Pre	5.77 ± 0.62	6.50 ± 0.54	0.12
	Post	5.33 ± 0.45	6.22 ± 0.53	
	Change	−0.44 ± 0.28	−0.27 ± 0.13	
	*p* value^a^	0.13	0.59	
Depression	Pre	2.61 ± 0.14	3.77 ± 0.53	0.10
	Post	2.38 ± 0.20	4.11 ± 0.59	
	Change	−0.22 ± 0.10	0.33 ± 0.25	
	*p* value^a^	0.12	0.21	
Total	Pre	30.44 ± 1.60	30.33 ± 1.34	< 0.001
	Post	18.94 ± 0.95	33.05 ± 2.14	
	Change	−12.11 ± 1.48	3.55 ± 1.39	
	*p* value^a^	< 0.001	0.06	

*Note:* The data were reported as mean ± standard error. Change calculated as follows: (after-before intervention) in each study group. *p* values of ≤ 0.05 were considered statistically significant.

Abbreviations: GHQ, general health questionnaire; HADS, hospital anxiety depression scale.

^a^Obtained from the paired sample *t*-test.

^b^Obtained from independent sample *t*-tests.

## Data Availability

The data will be available from the corresponding author upon request.
